# *In vivo* method to evaluate volumetric changes in bioceramic repair materials

**DOI:** 10.1590/0103-6440202405960

**Published:** 2024-09-16

**Authors:** Danilo Cassiano Ferraz, Jader Camilo Pinto, Juliane Maria Guerreiro-Tanomaru, Mario Tanomaru-Filho

**Affiliations:** 1 Department of Restorative Dentistry, São Paulo State University (UNESP), School of Dentistry, Araraquara, SP, Brazil.

**Keywords:** Calcium silicate, endodontics, x-ray microtomography

## Abstract

This study aimed to evaluate the effect of *in vitro* immersion solutions or an *in vivo* method on volumetric change of bioceramic root repair materials: Bio-C Repair (BCR, Angelus, Londrina, PR, Brazil) and Biodentine (BIO, Septodont, Saint-Maur-des-Fossés, France) compared to IRM (Dentsply Sirona, York, Pennsylvania, USA) by using microcomputed tomography (µCT) assessment. Tubes of polyvinyl chloride (PVC, 4 mm of length x 1.3 mm of inside diameter, n = 7) were filled with the materials for volumetric analysis in µCT. Samples were scanned after materials setting and after immersion in distilled water, PBS, or *in vivo* tissue fluid of subcutaneous tissue of rats for 7 days. IRM showed higher volumetric change than BCR and BIO in all immersion solutions (P<0.05). BIO and BCR presented similar volumetric changes when immersed in PBS and distilled water (P>0.05). When the *in vivo* method was used, BIO and BCR showed lower volumetric change (P<0.05), including an increase in volume for BCR. The immersion solutions influenced the evaluation of the volumetric change of bioceramic repair materials. Bioceramic materials show greater volumetric stability when evaluated by the *in vivo* method. The *in vivo* method in the subcutaneous tissue of rats can be an alternative for analyzing the properties of bioceramic cement, showing similarity with the clinical application.

## Resumo

O objetivo deste estudo foi avaliar o efeito de soluções de imersão ou de um método *in vivo* na alteração volumétrica dos materiais biocerâmicos reparadores Bio-C Repair (BCR, Angelus, Londrina, PR, Brasil) e Biodentine (BIO, Septodont, Saint-Maur-des-Fossés, França) em comparação com IRM (Dentsply Sirona, York, Pensilvânia, EUA), usando a avaliação por microtomografia computadorizada (µCT). Tubos de cloreto de polivinila (PVC, 4 mm de comprimento x 1,3 mm de diâmetro interno, n = 7) foram preenchidos com os materiais para análise volumétrica no µCT. As amostras foram escaneadas após a presa dos materiais e após a imersão em água destilada, PBS ou no tecido subcutâneo de ratos por 7 dias. O IRM apresentou uma alteração volumétrica maior do que o BCR e BIO em todas as soluções de imersão (P <0,05). BIO e BCR apresentaram uma alteração volumétrica semelhante quando imersos em PBS e água destilada (P> 0,05). Quando o método *in vivo* foi usado, BIO e BCR apresentaram uma menor alteração volumétrica (P <0,05), incluindo um aumento no volume para BCR. As soluções de imersão influenciaram a avaliação da alteração volumétrica dos materiais biocerâmicos reparadores. Os materiais biocerâmicos mostram maior estabilidade volumétrica quando avaliados pelo método *in vivo*. O método *in vivo* no tecido subcutâneo de ratos pode ser uma alternativa para analisar as propriedades de cimentos biocerâmicos, mostrando semelhança com a aplicação clínica.

## Introduction

Root repair materials play an essential role in vital pulp capping, regenerative endodontic therapy, perforation repair, and root-end filling due to their biocompatibility, bioactivity, and proper sealing ^1^. These materials are composed of calcium silicates and are known as bioceramic materials demonstrating biocompatibility and bioactivity ^2^. Biodentine (Septodont, Saint Maur des Fossés, France) and Bio-C Repair (Angelus, Londrina, PR, Brazil) are tricalcium silicate-based materials available in powder/liquid and ready-to-use compositions, respectively. Both have adequate biological properties ^3^ ^,^ ^4^ ^,^ ^5^.

Standards such as ISO 6876:2012 ^6^ and ANSI/ADA nº 57 ^7^ provide protocols for testing the physical properties of endodontic materials. Solubility can be related to microleakage and is evaluated by the difference in mass before and after immersion in distilled water ^8^. Dimensional change is assessed by linear measurement pre- and post-immersion in distilled water and can be related to the expansion or shrinkage of materials ^6^ ^,^ ^7^ ^,^ ^9^. Micro-computed tomography (µCT) has enhanced conventional tests by enabling non-destructive three-dimensional analysis and volumetric assessment ^10^. Volumetric analysis (in mm³) is conducted through µCT assessment and has been widely used ^9^ ^,^ ^11^ ^,^ ^12^. This approach allows for the correlation of volumetric changes with the material’s solubility in a single analysis ^13^.

Moreover, bioceramic materials may be affected by immersion solutions, since they are hydrophilic materials ^14^. Saline solutions such as PBS have been proposed as an alternative solution ^6^ ^,^ ^9^, with observed apatite deposition capacity *in vitro* ^15^. Moreover, a decrease in the solubility of root repair materials is observed when immersed in PBS ^4^ ^,^ ^16^. This variability in results may be related to the lack of specific guidelines for calcium silicate materials. In an attempt to carry out evaluation tests in clinical conditions, the investigation of physicochemical properties in *in vivo* models has been suggested ^17^. ISO 7405 standard ^18^ recommends tissue response evaluation of root repair materials by implanting the samples in the subcutaneous tissue of rats ^19^. The evaluation of bioceramic materials in rat subcutaneous tissue provided information on tissue compatibility, modulation of inflammation, and cytokine production ^20^ ^,^ ^21^. However, other properties such as porosity and dentin/material interface of bioceramic materials can be evaluated using a model with dentin tubes in rat subcutaneous tissue ^22^.

The evaluation of volumetric stability in different experimental models, including the *in vivo* model in rats, can provide important information on the behavior of materials for clinical application. This study aimed to evaluate the influence of different *in vitro* immersion solutions (distilled water, PBS) or an *in vivo* method on the volumetric change of bioceramic root repair materials. The null hypothesis was that there would be no difference among (a) immersion solutions and (b) the materials evaluated.

## Material and methods

### Design study

All experimental procedures were based on ARRIVE (Animal Research: Reporting of In Vivo Experiments) guidelines. All animal procedures were conducted by approval of the Ethics Committee on the Use of Animals of the São Paulo State University (CEUA/UNESP-FOAr) under protocol number 31/2020. Animal welfare was ensured by the ethical guidelines established by CEUA/UNESP-FOAr and the Normative Resolutions of the National Council for the Control of Animal Experimentation (CONCEA). Sample size calculation was performed using G*Power 3.1.7 software (Heinrich-Heine-Universität, Düsseldorf, Germany). One-way ANOVA was used with α error probability = 0.05, and power (1-β error probability) = 0.80. The size of specific effects for each variable was calculated from a previous study ^15^. Seven specimens per group were indicated.

### Sample preparation

The materials Biodentine (BIO) and Bio-C Repair (BCR) were handled according to manufacturer instructions in laminar flow. Flexible tubes of polyvinyl chloride (PVC) from scalp intravenous catheter with sizes of 4 mm and 1.3 mm of inside diameter (n=7) were filled with the materials. BCR was inserted with applicator tips supplied by the manufacturer, while BIO and IRM were inserted by pressuring the tube against material in a glass plate. The composition and manufacturer information of the materials are listed in Box 1.

**Box 1:**
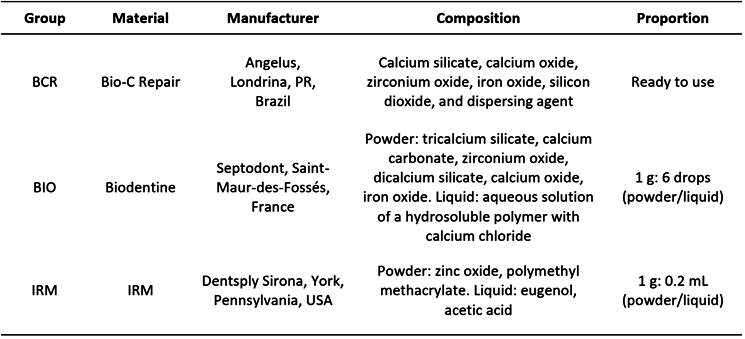
Tested materials

### Animal selection

Six male Holtzman rats (*Rattus norvegicus albinus*), aged 6-8 weeks and weighing approximately 280 g each, were selected. All animals were kept in polypropylene cages under controlled conditions (with a constant temperature of 22 ± 2ºC and relative humidity of 55 ± 10%) in a 12:12 hour light-dark cycle. Food and water were provided *ad libitum*. The animals were distributed into 3 groups (n=7) according to the tested materials.

### Volumetric assessment in subcutaneous tissue of rats

After filling, PVC tubes were scanned by µCT (SkyScan 1176; Bruker-micro-CT, Kontich, Belgium). The specimens were irradiated by UV light and immediately implanted into the dorsal subcutaneous tissue. The animals were anesthetized with ketamine hydrochloride (80 mg kg^-1^ body weight, Virbac do Brasil Indústria e Comércio Ltda., São Paulo, SP, Brazil) and xylazine hydrochloride (4 mg kg^-1^ body weight; União Química-Farmacêutica Nacional S/A, São Paulo, SP, Brazil), administered into the peritoneum. A 2 cm incision was made with a #15 scalpel blade (Fibra Cirúrgica, Joinville, Santa Catarina, Brazil). Four tubes were randomly allocated per animal. After 7 days, the animals were euthanized by anesthetic overdose, the implanted tubes were removed and scanned again at µCT.

The scanning parameters were: 80 kV voltage, 310 µA current, 8,74 µm voxel size, copper and aluminum (Cu + Al) filter, frame 4, step rotation 0.5º, and 180º rotation. The reconstruction of images was performed using NRecon software (V1.6.10.4; Bruker-micro-CT). Correction of beam hardening, artifacts, and smoothing were defined for each material. The images obtained were overlapped on the different periods using Data Viewer software (V1.5.2.4; Bruker-micro-CT). The total volume (mm³) of each cement was obtained by CTAn software (V1.15.4.0; Bruker-micro-CT). In CTAn, the tubes were divided in the middle, then the superior and inferior part was analyzed independently. The volumetric change between the baseline and the experimental period was calculated as follows: $\%VC=\left(\frac{V_{F\;x\;100}}{V_{I}}\right)$ , were V_F_ = final total volume and V_I_ = initial total volume.

Three-dimensional images of each group using CTVox software (V2.3.1.0; Bruker-micro-CT) were obtained. All procedures were repeated for samples immersed in 7.5 mL of distilled water and PBS (1x, D1408, Dulbecco's Phosphate Buffered Saline, Sigma-Aldrich) at 37 ºC.

### Statistical analysis

Data were submitted to the Shapiro-Wilk normality test. Two-way ANOVA and Tukey tests were performed for comparisons among groups. The significance level was 5% for all analyses.

## Results

**Figure 1:**
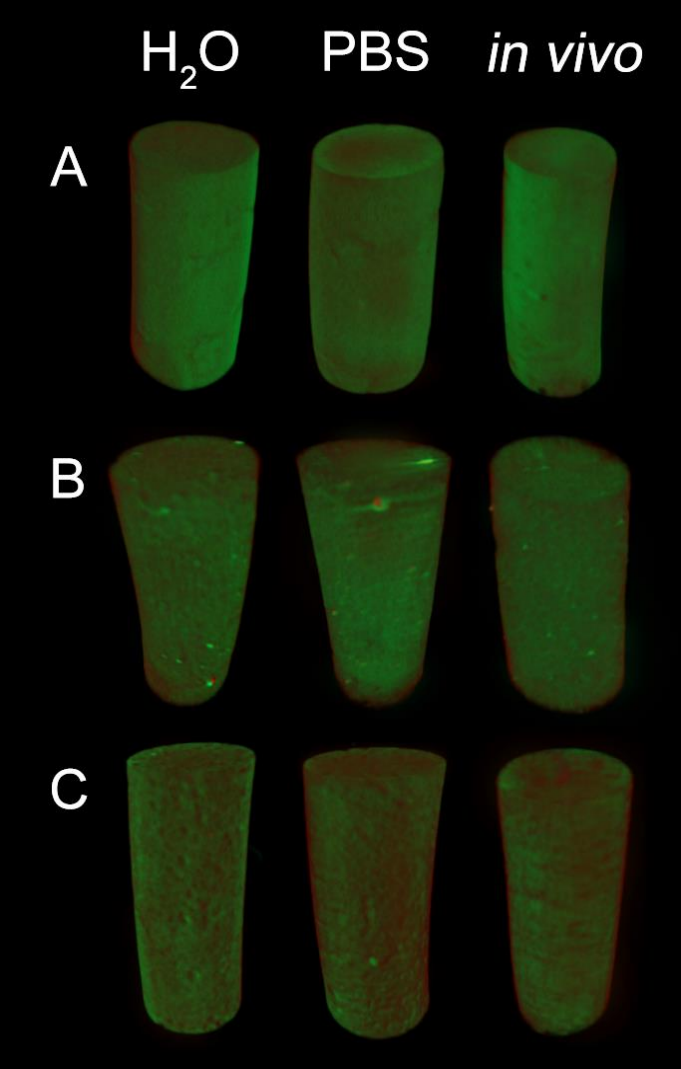
Three-dimensional µCT reconstructions showing overlapping of root repair materials before (red) and after (green) immersion for 7 days. Dense areas (green) indicate volume gain, while red areas indicate volume loss. A) BCR; B) BIO; and C) IRM.

Results regarding volumetric changes are presented in Table 1 and illustrated in Figure 1. IRM showed higher volumetric reduction than BCR and BIO in all experimental solutions (P<0.05). BIO and BCR presented similar volumetric changes when immersed in PBS and distilled water (P>0.05). A volumetric reduction was observed *in vivo* for BIO and IRM (P<0.05), whereas BCR showed an increase in volume in the same media.

**Table 1:** Mean and standard deviation of volumetric change percentual root repair materials after immersion in distilled water, PBS, or *in vivo* tissue fluid for 7 days.

	BCR	BIO	IRM
Distilled water	-0.262 ± 1.124^aB^	-0.316 ± 0.129^aB^	-0.475 ± 0.129^bA^
PBS	-0.311 ± 0.090^aB^	-0.268 ± 0.063^aB^	-1.183 ± 0.222^bC^
*in vivo*	0.196 ± 0.061^aA^	-0.075 ± 0.035^bA^	-0.868 ± 0.300^cB^

Different superscript lowercase letters in the same row indicate a significant difference among cements. Different superscript capital letters in the same column indicate a significant difference among immersion solutions (p<.05). Negative values of volume change represent volume loss, and positive values represent expansion.

## Discussion

This study assessed the volumetric change of two bioceramic materials compared with zinc oxide and eugenol-based material when immersed in distilled water, PBS, or *in vivo*, tissue method using fluid of subcutaneous tissue of rats. The null hypotheses were fully rejected, since in general (a) the immersion solutions affected the volumetric change of materials and (b) differences among materials were observed.

The use of rat subcutaneous tissue is an established methodology for biological tests ^22^ ^,^ ^23^. However, this is the first study to assess the effect of *in vivo* immersion on volumetric changes in calcium silicate materials. When immersed in *vivo* tissue fluid, BIO and BCR showed lower volumetric change, including an increase in volume for BCR. A previous study described that BCR is mostly composed of oxygen, carbon, zirconium, and calcium ^24^. The gain in volume for this material could be justified by the chemical reaction between calcium ions and carbon dioxide that leads to the formation of calcite crystals on the surface of the material ^23^.

BIO presented a reduction in volumetric loss when immersed in tissue fluid. The mechanism of apatite deposition presented by BIO in phosphate-like solutions might be increased when the material is used under *in vivo* conditions. The *in vivo* method can allow apatite deposition promoting volumetric stability to bioceramic materials. This apatite-deposition behavior has already been reported for bioceramic materials when immersed in PBS ^9^. However, *in vivo*, evaluation is closer to the clinical situation by allowing the interaction between calcium silicate-based materials with tissue fluid. All materials tested showed greater volumetric stability when using the *in vivo* method.

IRM is a zinc oxide-eugenol-based cement used as a comparative for root-end filling materials studies ^25^. In the present study, IRM showed higher volumetric change than BCR and BIO in all immersion solutions. Higher values of volumetric loss of IRM than BCR and BIO could be related to the leaching of eugenol ^16^. A µCT study showed similar volumetric change between BIO and IRM when immersed in PBS ^9^. A similar volumetric change was reported for BCR and BIO in a gypsum-based model when immersed in distilled water ^13^. Otherwise, it was reported lower volumetric change for IRM than BIO when immersed in distilled water using µCT assessment and samples with different dimensions ^12^. IRM presented less than 1% volume loss when immersed in distilled water and tissue fluid, which is similar to a previous study for IRM ^12^.

In the present study, BIO and BCR had similar volumetric changes when immersed in PBS and distilled water, and they showed values below 1%. Otherwise, BIO also showed a volumetric change of next to 2% when evaluated in PBS ^26^. However, a volumetric change near zero was observed for BIO in the present study when materials were immersed in the tissue fluid. Volumetric change above 1% by µCT assessment was observed for BCR in gypsum-based models when immersed in distilled water ^13^. In the present study, BCR showed less than 1% of volume loss in distilled water or PBS. Similar results were found recently for BCR when immersed in distilled water for 7 days ^27^. Meanwhile, BCR showed an increase in volume when immersed in tissue fluid.

The immersion solutions influenced the evaluation of the volumetric change of bioceramic repair materials. Bioceramic materials have greater volumetric stability when used by the *in vivo* method. The *in vivo* method in the subcutaneous tissue of rats can be an alternative for analyzing the properties of bioceramic materials, showing similarity with the clinical application.
